# The Intrauterine Device in Women with Diabetes Mellitus Type I and II: A Systematic Review

**DOI:** 10.1155/2013/814062

**Published:** 2013-12-11

**Authors:** Norman D. Goldstuck, Petrus S. Steyn

**Affiliations:** ^1^Department of Obstetrics and Gynaecology, Tygerberg Hospital, Western Cape 7505, South Africa; ^2^Reproductive Health and Fertility Regulation, Department of Obstetrics and Gynaecology, Groote Schuur Hospital and University of Cape Town, Cape Town 7925, South Africa

## Abstract

*Background*. Women with diabetes mellitus type I and type II need effective contraception for personal and medical reasons. Long acting reversible contraceptive (LARC) methods are among the most efficient and cost-effective methods. *Study Design*. We searched the Popline, PubMed, and clinicaltrials.gov databases from 1961 to March 2013 for studies on the efficacy of the IUD in diabetic women and the possible changes it may produce in laboratory parameters. Studies of at least 30 subjects with DM1 or DM2 who were studied for 6 to 12 months depending on the method of analysis were eligible. *Results*. The search produced seven articles which gave event rate efficacy evaluable results and three which evaluated the effect of the IUD on laboratory parameters. One of the earlier efficacy studies showed an abnormally high pregnancy rate which sparked a controversy which is discussed in the Introduction section. The remaining 6 studies produced acceptable pregnancy rates. The three laboratory studies showed that the copper and levonorgestrel releasing IUD/IUS do not affect the diabetic state in any way. *Conclusions*. The copper bearing and levonorgestrel releasing IUDs are safe and effective in women with diabetes type I and diabetes type II although the evidence in the latter is limited.

## 1. Introduction

Diabetes mellitus is a ubiquitous disease. The affluent world is engulfed in an epidemic of diabetes mellitus type II (DM2) [1]. Before the discovery of insulin, diabetes mellitus type I (DM1) sufferers died very rapidly. Historically, short life spans and inadequate nutrition made diabetes mellitus type II (DM2) very uncommon. Initially DM2 was termed “aged onset” or “maturity onset” diabetes indicating that it appeared in later life. This is no longer the case. It is now prevalent even in the young [1] and it will affect many women who are of reproductive age and who may want to use contraception. Many will be suited to and may want to use a long acting reversible contraceptive (LARC) method such as the intrauterine device (IUD).

During the late 1970s the possible problem of the IUD in Insulin dependent diabetes mellitus (IDDM) emerged. This was at a time when the concern was that IUDs could cause infection [2] and women with diabetes mellitus were also considered poor candidates for the combined oral contraceptives [3] that were available at the time. Diabetic women of reproductive age (almost exclusively with what is now termed DM1) were left with limited options as injectable contraception was not yet available in Europe and North America. This left only the progestin only pill (POP), mechanical methods, the rhythm method, and sterilisation for this group.

The problem of the IUD in diabetics began in Denmark with a report by Wiese and Osler in 1974 [4]. Wiese later reported different results [5]. Then in 1979 Steel and colleagues in Scotland reported that in their clinic 8 out of 22 IDDM IUD users had become pregnant [6] and afterwards reported that 11 out of 30 were now pregnant of which 5 were using the copper 7, 5 the Saf-T-Coil, and one a Dalkon Shield IUD [7]. They also showed that the copper containing IUDs of diabetic women (including those who did not become pregnant) had fewer calcareous deposits than in nondiabetics and that these deposits contained a higher level of sulphur and chloride than usually found in nondiabetics. The higher levels of sulphur and chloride were also found in nondiabetic women who became pregnant. They agreed with the earlier findings presented in a paper by Larsson in which he showed that there was reduced fibrinolytic activity in the endometrium in diabetic women using copper IUDs [8]. Fibrinolytic activity is due in part to prostaglandin synthetase activation which was thought to be required for the efficacy of the copper IUD. Its absence was thought to be a possible reason why copper IUDs were less effective in diabetics (and in nondiabetics who became pregnant). This did not of course explain the failures of the plastic IUDs like the Lippes Loop, Antigon, and Dalkon Shield.

This was not the experience in continental Europe where many reported observations found no evidence of problems in diabetic women who were using the IUD [9–14]. The Medical Advisory Panel of the Family Planning Association of the United Kingdom and that of International Planned Parenthood Federation met to debate the problem but decided that there was not enough evidence to issue a general recommendation to avoid using the IUD in diabetic woman [15].

A positive report from Oxford [17] in 1982 and other British and European authors [18–20] and finally a report from Israel [21] in 1985 that previous gestational diabetes might also be a contraindication to IUD use because of increased pregnancy problems ended this debate. That same year the article by Cramer at al. [22] on tubal infertility which showed that it was preceded mainly by plastic, not copper IUD use and later the findings of Farley et al. in 1992 [23] which showed that the IUD *per se* was not the cause of pelvic infections began to change the IUD landscape.

This paper evaluates the use of inert (plain plastic), copper bearing and hormone releasing IUDs in women with DM1 and DM2 and compares the pregnancy rate with nondiabetic experiences and with nondiabetic controls where available. Secondarily it compares the biochemical changes in copper IUD users and users of the levonorgestrel releasing IUS (Mirena) with those using a combined oral contraceptive pill (COC) or hormonal injection or hormonal releasing implant (Norplant).

## 2. Methods

We searched the Popline, PubMed, and clinicaltrials.gov databases from 1961 to March 2013. The terms used were as follows.

 (i) *Popline*: “Intrauterine device/Intrauterine system,” “IUD/IUS,” and “Diabetes mellitus.”
 (ii) *PubMed*:“Intrauterine device/Intrauterine system” and “IUD/IUS” + “Diabetes mellitus.”
 (iii) *clinicaltrials.gov*: “Intrauterine device,” “diabetes type I,” “diabetes type II,” and “gestational diabetes.”

Gestational diabetes was added to the clinicaltrials.gov search although only one previous article [21] was found on this topic because of the improved state of knowledge in this area and better ideas of its possible future significance.

References from cross references in listed papers were checked when they did not appear on any of the search databases. An attempt was made to contact authors of papers of obscure journals for further information if not listed in the previous databases.

Articles in all languages were acceptable but in some cases only the English abstracts were examined. The Chinese databases Wanfang Data or Weipu Data were not searched and there were no cross-references to any Chinese articles in the reference lists of any of the articles which were reviewed. Many of the references from the introductory section also came from this search.

Articles were qualified for the inclusion for investigation of IUD efficacy (pregnancy rates) and for IUD related problems (event rates for medical and tolerability problems) if they met the following criteria.

- Minimum of thirty subjects.
- At least one year of followup if results were expressed as a percentage or Pearl Index.
- Six months of followup if life table analysis was used.

Articles on the investigation of the possible role of the IUD on diabetes control required investigation of laboratory values for a minimum period of one year. Summary odds ratios were not calculated because of the variable methods of data reporting and some of the poorly quantifiable outcomes reported. The variable means of reporting over many years lead to disk of bias for that reason and the fact that failure to use life table analysis in any IUD study will almost certainly bias the results.

A printout of lists of studies from all the databases was taken to eliminate duplicates and determine if the studies met the inclusion criteria. The initial search was conducted by one author (NDG) and the extraction and analysis by both authors.

Many changes have taken place in the understanding and treatment of diabetes mellitus over the last thirty or more years, including changes in terminology.

The terms insulin dependent diabetes mellitus (IDDM) and noninsulin dependent diabetes mellitus (NIDDM) are no longer used. These terms are not interchangeable with DM1 and DM2 necessitating certain assumptions with regard to interpretation of the data and this is explained where applicable.

## 3. Results

**Figure 1:**
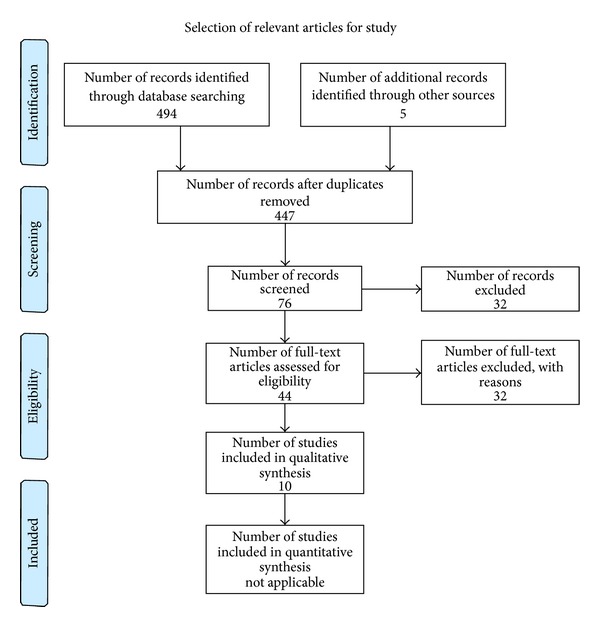
Flowchart of collection and synthesis of data. (PRISMA) [16].

The search identified 499 articles of which seven met the inclusion criteria for the evaluation of IUD efficacy in the diabetic group [5, 7, 24–29]. Three articles met the criteria for the evaluation of laboratory parameters in diabetic women while using an IUD [30–32]. The disposition of potential papers is given as a flow chart (Figure 1). The 32 full-text articles which were excluded (see flow diagram) were excluded because they did not meet the inclusion criteria because these articles were predominantly concerned with all types of contraception not just IUDs in diabetic women. There was some overlap in that studies reporting efficacy of the IUD also had biochemical reports (and information on other IUD related concerns, e.g., removal for pain and/or bleeding and infection rates and continuation rates) and the laboratory studies made note of pregnancy occurrences (or lack thereof).

The essential data from the efficacy studies is presented in Table 1 and the data from the biochemical studies is presented in Table 2. There was only one efficacy study in women with DM2 and two of the three laboratory studies included subjects with DM2.

There was one study listed in clinical trials.gov and is a prospective study of women with gestational diabetes who will be randomly allocated to a levonorgestrel IUD (LNG-IUD) or copper IUD (Paragard-TCu380A) as postpartum contraception.

In some of the older studies the diabetes is described as IDDM and NIDDM. For practical purposes it can be assumed that the IDDM patients were DM1 since the women were younger and most young patients with DM2, especially then, would not have been using insulin. The NIDDm patients are more difficult to quantify. Most probably had DM2 but it is likely that some at least were women with previous gestational diabetes. This was not stated explicitly.

The rigid quantification of diabetes in 1970s and 1980s was not as specific as it is today for many reasons including the nonavailability of routine serum insulin evaluations, glycosalated haemoglobin, and other biochemical tests. Six of the seven efficacy studies were prospective and one was a retrospective questionnaire study. Three of the efficacy studies were controlled against a nondiabetic group of users of similar types of IUD. The study using the Antigon IUD [4] had a high expulsion rate in both the diabetic and nondiabetic subjects which was a particular problem for this type of device. The results show that pregnancy rates are acceptable varying from 0.3 to 4 at one year except for the Gosden [7] study which is a clear outlier. The pregnancy rates are similar but not comparable because of the different methods of calculation.

## 4. Discussion

Historically the modern use of the IUD in Europe and North America has faced an uphill struggle for acceptance since it was introduced by Ernst Grafenberg in Germany and brought to the United States in the early twentieth century. The Lippes Loop and other plastic IUDs were introduced in the early sixties and all had their detractors. Historically the IUD has been involved in numerous controversies relating to its use. There have been numerous contraindications to its use including nulliparity, potential for inducing infections, and even the possibility of causing endometrial cancer. At the time it was feared that diabetes may also become a new contraindication to IUD use. Neither diabetes nor any of the other potential problems remains as a barrier to the IUD as the LARC method and emphasizes the necessity for carefully designed and conducted studies before it is possible to come to reasonable evidence based scientific conclusions. The authors of the early diabetic pregnancies report maintained that as most of the pregnancies had occurred soon after the device was inserted that more insertions would only lead to an even higher pregnancy rate [3, 7]. The need for blinded or at least controlled studies where blinding is impossible as in IUD studies is imperative to avoid rushing to early and sometimes incorrect judgments.

The concept of life table analysis for IUDs had already been introduced by Tietze [33] and there remains no substitute for life table analysis in IUD evaluation. There was also no explanation of the mechanism of increased fibrinolysis in the endometrium of diabetic copper IUD users or why these users should deposit increased sulphur and chloride on the threads of the devices they examined. There was no evidence presented that it was related to glucose levels or glycosalated haemoglobin or lipid changes. It is reasonably certain that current guidelines for conducting and publishing clinical research, for example, “level of evidence” and “risk of bias” would probably have prevented this unfortunate error in the role of the IUD as a contraceptive in diabetic women.

Currently the LARC methods are the methods which are most able to reduce the unwanted pregnancy rates [34]. Women with DM1 and DM2 may have increased problems during pregnancy and therefore want to avoid unintended pregnancies. This systematic review confirms that there are sufficient well controlled studies to conclude that both the copper bearing and levonorgestrel IUDs are effective and safe for women with DM1 or DM2.

The increase in prevalence of DM2 in the younger age groups and in young women of reproductive age makes it important to ascertain the efficacy and safety of the IUD in this group. There is only one study of the copper IUD in this group. Confirmation of these results in general and for other types of copper bearing devices and the LNG-IUS would be welcome although it is not likely that the outcomes would be different. There are no studies as yet of the efficacy and other event rates with the LNG-IUS in DM1 or DM2. The LNG-IUS has only been evaluated for its possible effects on diabetes metabolism in DM1 and to a lesser extent DM2.

The World Health Organisation (WHO) assigns a category rating of 1 for the use of copper IUDs in diabetes and a category 2 rating for the levonorgestrel IUD in diabetes [32, 35]. A call for the liberalisation of this IUD to category 1 has not been heeded [32]. An efficacy study of this device in DM1 and DM2 women could help it to gain category 1 status provided it produced no adverse effects in these users.

## 5. Conclusion

Current copper bearing and hormonal IUDs are both effective and safe for use as LARC methods of contraception for diabetic women. While the levonorgestrel IUD does not produce metabolic changes in DM1, it has not yet been adequately studied in DM2. Demographics suggest that young women with DM2 could become important candidates for intrauterine contraception.

## Figures and Tables

**Table 1:** Pregnancy rates in diabetic women using the IUD.

Study	Subjects (*N*)	Type of IUD	Type of diabetes (DM1or DM2) (IDDM or NIDDM)	Pregnancy rate at 1 year	Final pregnancy rate (end of study)	Comments
Wiese [5]	118	Antigon I, II, III, IV, F	IDDM	4.8 ± 2.4SE^◊^	3.5 ± 2SE^◊^ (24 months)	High removal and expulsion rate, similar in normal subjects
Buchsenschutz [24] and Serfaty [25]	56	CuT200, Cu-7, MLCu250	IDDM and NIDDM	0	1.7% (36 months)	Noncumulative pregnancy rate, salpingitis 3.6% and expulsion 10.1%
Gosden et al. [7]	30	Cu-7, Saf-T-Coil, Dalkon Shield	IDDM	46%	46% (12 months)	Abnormal chloride and sulphur deposits on the copper devices
Skouby et al. [26]	103	CuT200	IDDM	1.0^◊^	1.0^◊^ (12 months)	Corrosion surfaces of copper-no differences between diabetic and non-diabetic controls
Kimmerle et al. [27]	59	Cu-Safe300 (Flexi-T300)	DM1	1.7^†^	2.6^†^ (36 months)	Same rate as controls, 78% nulliparous, average HbA1c 7%
Kimmerle et al. [28]	127	Copper IUDs-not specified	DM1	Not available	0.3^†^ (60 months)	Retrospective study, 70% nulliparous, average HbA1c 8 ± 1.7%, controlled
Kjos et al. [29]	117	TCu380A	DM2	0.9^◊^	4.0^◊^ (36 months)	100% multiparous, open study

IDDM: insulin dependent diabetes mellitus; NIDDM: non-insulin dependent diabetes mellitus; DM1: diabetes mellitus type I; DM2: diabetes mellitus type II; ^◊^life table data; ^†^Pearl Index; HbA1c: glycosylated haemoglobin.

**Table 2:** Laboratory values in diabetic women using the IUD.

Type of diabetes	Reference	Subjects (*n*)	IUD type	Mean initial and concluding values (12 months)			Comments
				HbA1c (%)	IR (U)	FBG	
DM1	Grigoryan et al. [30]	11	TCu380A	7.8-7.8	64.5-64.6^*◊*^		Perimenopausal subjects, no lipid or coagulability changes versus COC; TCu380A was control
	Grigoryan et al. [30]	11	LNG-IUS	7.6-7.7			
	Diab and Zaki [31]	15	Tcu380A	7.1 ± 0.16^†^		109–99^*∙*^	No lipid or coagulability changes versus Norplant, COC and DMPA; TCu380A was control
	Rogovskaya et al. [32]	29	LNG-IUS	5.6–6.3	35.2-35.1	5.2–7.4^o^	No changes in HbA1c and FBG for both IUDs. The TCu380A acted as control. There were no pregnancies.
	Rogovskaya et al. [32]	30	TCu380A	5.5–6.3	37.3–37.1	5.0–7.5^o^	

DM2	Grigoryan et al. [30]	11	TCu380A	7.5-7.4	46 ± 10.7^◊^		Four perimenopausal DM2 on insulin. No laboratory changes as for DM1
	Grigoryan et al. [30]	11	LNG-IUS	7.4–7.6			
	Diab and Zaki [31]	5	TCu380A			109–99^*∙*^	

HbA1c: glycosalated haemoglobin; FBG: fasting blood glucose; IR (U): insulin requirement (international units); COC: combined oral contraceptive; DMPA: depomedroxyprogesterone acetate; ^*∙*^mg/dL; ^o^mmol/L; ^*◊*^pooled values TCu380A and LNG-IUS; ^†^Initial value for DM1 and DM2 (16 subjects).
